# The Splicing Efficiency of Activating *HRAS* Mutations Can Determine Costello Syndrome Phenotype and Frequency in Cancer

**DOI:** 10.1371/journal.pgen.1006039

**Published:** 2016-05-19

**Authors:** Anne-Mette Hartung, Jeff Swensen, Inaki E. Uriz, Morten Lapin, Karen Kristjansdottir, Ulrika S. S. Petersen, Jeanne Mari V. Bang, Barbara Guerra, Henriette Skovgaard Andersen, Steven F. Dobrowolski, John C. Carey, Ping Yu, Cecily Vaughn, Amy Calhoun, Martin R. Larsen, Lars Dyrskjøt, David A. Stevenson, Brage S. Andresen

**Affiliations:** 1 Department of Biochemistry and Molecular Biology and The Villum Center for Bioanalytical Sciences, University of Southern Denmark, Odense M, Denmark; 2 Caris Life Sciences, Phoenix, Arizona, United States of America; 3 Department of Pathology, Children's Hospital of Pittsburgh, Pittsburgh, Pennsylvania, United States of America; 4 ARUP Laboratories, Salt Lake City, Utah, United States of America; 5 Department of Pediatrics, University of Utah, Salt Lake City, Utah, United States of America; 6 Department of Pediatrics, University of Minnesota, Minneapolis, Minnesota, United States of America; 7 Department of Molecular Medicine, Aarhus University Hospital, Aarhus, Denmark; 8 Division of Medical Genetics, Stanford University, Stanford, California, United States of America; University of Oxford, UNITED KINGDOM

## Abstract

Costello syndrome (CS) may be caused by activating mutations in codon 12/13 of the *HRAS* proto-oncogene. *HRAS* p.Gly12Val mutations have the highest transforming activity, are very frequent in cancers, but very rare in CS, where they are reported to cause a severe, early lethal, phenotype. We identified an unusual, new germline p.Gly12Val mutation, c.35_36GC>TG, in a 12-year-old boy with attenuated CS. Analysis of his *HRAS* cDNA showed high levels of exon 2 skipping. Using wild type and mutant *HRAS* minigenes, we confirmed that c.35_36GC>TG results in exon 2 skipping by simultaneously disrupting the function of a critical Exonic Splicing Enhancer (ESE) and creation of an Exonic Splicing Silencer (ESS). We show that this vulnerability of *HRAS* exon 2 is caused by a weak 3’ splice site, which makes exon 2 inclusion dependent on binding of splicing stimulatory proteins, like SRSF2, to the critical ESE. Because the majority of cancer- and CS- causing mutations are located here, they affect splicing differently. Therefore, our results also demonstrate that the phenotype in CS and somatic cancers is not only determined by the different transforming potentials of mutant *HRAS* proteins, but also by the efficiency of exon 2 inclusion resulting from the different *HRAS* mutations. Finally, we show that a splice switching oligonucleotide (SSO) that blocks access to the critical ESE causes exon 2 skipping and halts proliferation of cancer cells. This unravels a potential for development of new anti-cancer therapies based on SSO-mediated *HRAS* exon 2 skipping.

## Author Summary

*HRAS* was the first human proto-oncogene reported and p.Gly12Val in codon 12 the first oncogenic mutation described. Somatic mutations in *HRAS* are important drivers in cancer, and germline mutations cause Costello syndrome. Until now, it has been believed that the severity of mutations located in the *HRAS* codon 12/13 mutational hot spot sequence is exclusively determined by the activity of the encoded proteins. Here we show that mutations in exon 2 of the *HRAS* proto-oncogene can have a previously unrecognized effect on splicing efficiency and thereby determine HRAS activity. We report a patient with Costello syndrome (CS), who despite having the classical, severe, oncogenic p.Gly12Val mutation in *HRAS* has a mild clinical phenotype. We show that this is due to his specific sequence change, c.35_36GC>TG, encoding p.Gly12Val, which disrupts an ESE fundamental for efficient splicing of exon 2. We have explored this in detail and were able to show that *HRAS* exon 2 is a weak exon, which is dependent on the balance between positive and negative splicing regulatory factors, like SRSF2 and hnRNPF/H in order to be properly included. We show that different mutations in the mutational hot spot in *HRAS* codon 12 and 13 (c.34-39) affect splicing of *HRAS* differently, suggesting that this mechanism may also influence their occurrence in CS and their oncogenic potential in somatic cancers. Finally, we show that blocking access to the fundamental ESE, located in *HRAS* c.34-39, using a splice switching oligonucleotide (SSOs), causes exon 2 skipping, abolishes production of functional HRAS protein and halts proliferation of cancer cells. This shows a previously unknown weakness of the *HRAS* proto-oncogene, namely that exon 2 is weakly defined and therefore is a suitable target for SSO-based therapy, thereby pointing towards a potential new direction for therapeutic targeting of RAS in anti-cancer treatment.

## Introduction

The Harvey rat sarcoma viral proto-oncogene homolog (*HRAS*) was the first human proto-oncogene to be identified [1]. The HRAS protein is a GTPase, which mediates signal transduction from growth factor receptors important for cellular proliferation, growth and survival. Somatic mutations in *HRAS* are present in many cancers and the vast majority of mutations affect codons 12 and 13 (c.34-39) leading to a constitutively active protein. The c.35G>T mutation (p.Gly12Val) in *HRAS* was the first mutation in a proto-oncogene that was implicated in cancer [2,3] and it is the second most frequently reported *HRAS* mutation in human cancers (Cosmic database: http://cancer.sanger.ac.uk/cosmic/gene/overview?ln=HRAS). The p.Gly12Val mutant has the lowest GTPase activity [4] and highest transformation potential among *HRAS* mutants [5,6].

Germline mutations in *HRAS* cause Costello syndrome (CS) (MIM: 218040), which is a congenital disease characterized by postnatal growth retardation, short stature, tumor predisposition, developmental delay, and abnormalities of the heart (cardiomyopathy), skin and skeletal muscles [7].

The vast majority (75%) of CS is caused by heterozygous c.34G>A (p.Gly12Ser) activating mutations in *HRAS* [8]. Heterozygous c.35G>C (p.Gly12Ala) and c.37G>T (p.Gly13Cys) *HRAS* mutations are also frequent in CS patients, making up 10% and 7% of alleles, respectively [8]. Whereas these mutations result in a relatively homogenous phenotype, a particularly severe, early lethal form of CS has been observed in a few patients with the less frequently observed p.Gly12Val (encoded by c.35G>T, c.35_36GC>TT or c.35_36GC>TA), p.Gly12Asp (c.35G>A) or p.Gly12Cys (c.34G>T) mutations [9–12]. These severe CS phenotypes are consistent with the higher transforming potential of the p.Gly12Val, p.Gly12Asp and p.Gly12Cys mutant proteins [5] and are also reflected in the higher frequencies of these mutations in cancer (Cosmic).

So far it has therefore been believed that the relative frequency of the different *HRAS* mutations in cancer and CS simply reflects differences in the oncogenic potential of the encoded proteins and differences in the rate they occur by spontaneous mutations. Recently, it was, however, demonstrated that a phenomenon termed “selfish selection” may be an important factor determining the mutation spectrum observed in CS. Selfish selection both offers an explanation for the puzzling fact that CS has an extreme paternal bias in origin and occurs with a frequency two—three orders of magnitude higher than expected from the background mutation rate [8]. This is explainable by the fact that *HRAS* mutations offer different selection advantages in male germ cells depending on their oncogenic potential, and that their proportion increases with paternal age dependent on the selective advantage.

Exonic mutations may, however, also have other effects than the expected changes in the protein, which can be predicted based on the resulting change from one codon to another according to the genetic code. A so called “splicing code” [13], which predicts that exonic mutations may have effects by impacting splicing regulatory elements, is beginning to emerge as an important cause of human disease, including cancer. This was elegantly demonstrated in a study by Supek and co-workers, who showed that synonymous mutations frequently act as driver mutations in cancer by altering exonic splicing regulatory motifs [14]. In particular, it was demonstrated that cancer-associated synonymous mutations frequently create exonic splicing enhancers (ESEs) and destroy exonic splicing silencers (ESSs) which regulate oncogene splicing in tumors. Additionally, expression and activity of splicing regulatory factors, like the SR-proteins and the hnRNP proteins are often dysregulated in cancer leading to aberrant splicing of oncogenes and tumor suppressor genes. A prominent example is the SRSF2 splicing regulatory factor, which is frequently mutated to a dominant negative form in myelogenic diseases [15]. Other important splicing regulatory factors such as SRSF1, SRSF3 and SRSF6 have been described as potent oncogenes [16–18], underscoring the importance of dysregulated splicing in cancer.

Here we demonstrate for the first time that *HRAS* exon 2 is a vulnerable exon, which is dependent on binding of splicing regulatory proteins to ESEs in order to be correctly included in the *HRAS* mRNA. Importantly, we show that a mutation in the codon for Glycine 12 (c.34-39) can abrogate formation of constitutively active p.Gly12Val HRAS in a CS patient by mediating pronounced exon 2 skipping. Moreover, we show that this vulnerability of *HRAS* exon 2 splicing can be exploited by employing splice switching oligonucleotides (SSOs) to induce *HRAS* exon 2 skipping. This provides proof of principle for a new mechanism for knocking out oncogenic HRAS, which may be used therapeutically to treat cancer.

## Results

**Fig 1:**
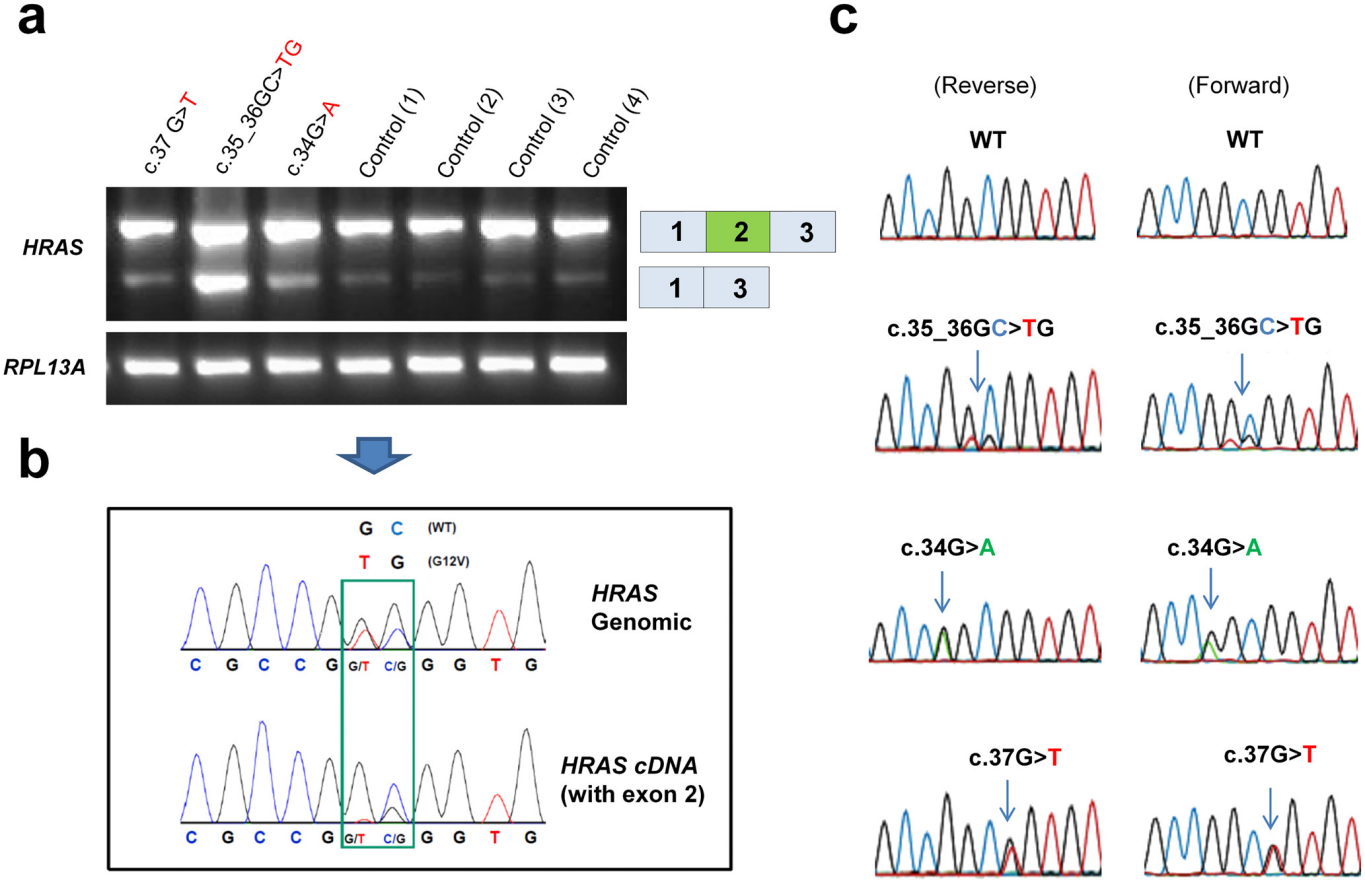
Analysis of patient DNA and cDNA. (**a**) RT-PCR analysis of lymphocyte cDNA from four unrelated controls and three individuals with Costello syndrome (CS), caused by heterozygosity for c.34G>A, c.37G>T and c.35_36GC>TG (index individual), revealed pronounced exon 2 skipping in the index individual. The low levels of exon 2 skipping observed in controls and individuals with CS with other genotypes indicates that exon 2 is inefficiently spliced. RPL13A was amplified as a control. (**b**) Comparison of sequence analysis of genomic DNA and lymphocyte cDNA from the index individual, shows that wild type and c.35_36GC>TG alleles are equally present in genomic DNA, but not in full length cDNA. (**c**) Chromatograms from sequencing of the full length band (exon 2 inclusion) from lymphocyte cDNA in forward and reverse direction from individuals with CS heterozygous for c.35_36GC>TG, c.34G>A and c.37G>T.

We investigated a 12-year-old boy with an attenuated CS phenotype (see online methods). Sequence analysis of his DNA from multiple tissues showed a heterozygous c.35_36GC>TG germline mutation that was predicted to result in p.Gly12Val (Fig 1) There was no evidence of mosaicism (S1 Fig). Presently only a few individuals with CS with a p.Gly12Val mutation (c.35G>T, c.35_36GC>TT, c.35_36GC>TA) have been reported and all had a very severe clinical presentation, with death typically within the first months of life [9,10].

Because mosaicism did not explain the mild clinical presentation of the severe p.Gly12Val mutation in this boy, RNA from lymphocytes was examined and showed extensive exon 2 skipping, and showed that the c.35_36GC>TG mutation was nearly absent in correctly spliced *HRAS* mRNA (Fig 1). Low levels of exon 2 skipping were also observed in cells from controls and individuals with CS heterozygous for other *HRAS* mutations, indicating that exon 2 may be difficult to splice efficiently. Consistent with this, different human tissues also show low levels of *HRAS* exon 2 skipping and the amount varies between tissues, possibly reflecting tissue specific differences in the splicing regulatory factors which mediate exon 2 inclusion (S2 Fig). An mRNA without exon 2 will not produce a functional protein as the switch domains, switch I (amino acids 32–38) and switch II (amino acids 59–67), are fundamental for RAS-GTP/GDP binding, and thus for biological function. Exon 2 (amino acids 1–37) encodes a major part of the switch I domain. In particular threonine 35, which binds the terminal phosphate (γ-phosphate) of GTP in the active site, is encoded by exon 2. Therefore, if a protein were to be produced from an mRNA lacking exon 2 it would not be functional. Additionally, the normal ATG start codon is located in exon 2. A potential alternative in-frame ATG start codon is located in exon 3, but it’s use would produce a protein with a deletion of 66 amino acids from the amino terminal end, and thereby exclude both switch I and switch II. Consequently, loss of HRAS activity due to exon 2 skipping from the c.35_36GC>TG mutation can explain the attenuated phenotype in the individual with CS.

**Fig 2:**
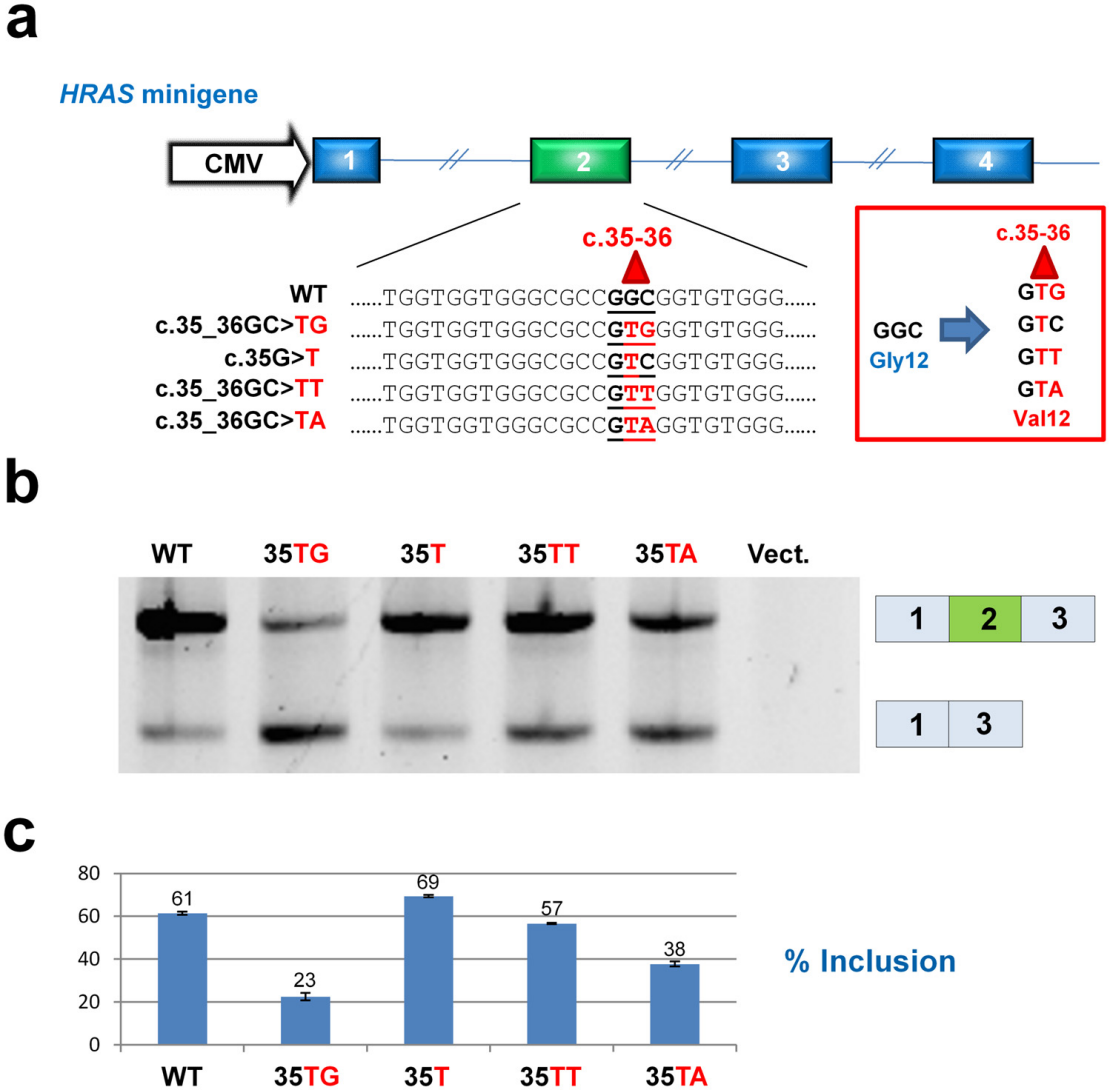
p.Gly12Val mutations in codon 12 of *HRAS* exon 2 affect splicing differently. (**a**) Displays the *HRAS* minigene construct and the wild type and mutant sequences. The *HRAS* minigene consisted of the first four *HRAS* exons (including the natural intronic sequences) cloned into the polylinker of a pcDNA3.1+ vector. (**b**) Representative results from HepG2 cells transfected with wild type and mutant minigenes. Splicing analysis by PCR amplification and agarose gel electrophoresis reveals extensive exon 2 skipping from c.35_36GC>TG construct and moderate exon 2 skipping from c.35_36GC>TA construct. The lane labelled “Vect.” shows the results from a sample transfected with an empty p.cDNA3.1+ vector. (**c**) Quantification of the exon 2 inclusion rate from triplicate transfections using a fragment analyzer. Numbers are % inclusion. Calculations are based on molar ratios.

Since individuals with CS with other *HRAS* mutations, c.35G>T, c.35_36GC>TT and c.35_36GC>TA encoding the p.Gly12Val mutant protein have suffered from a very severe CS phenotype, we investigated the effect of these mutations on *HRAS* exon 2 splicing using a *HRAS* minigene. Transfection of the wild type and mutant minigenes into HepG2 cells confirmed that the c.35_36GC>TG mutation by itself causes high levels of exon 2 skipping, whereas the c.35_36GC>TT and c.35_36GC>TA p.Gly12Val mutations cause a low or modest increase in exon 2 skipping, respectively (Fig 2). The severe p.Gly12Val, c.35G>T mutation did not increase exon 2 skipping. Taken together, these data show that different nucleotide changes in codon 12 (c.34-36) of *HRAS* exon 2 can regulate HRAS activity by affecting the efficiency of *HRAS* exon 2 inclusion into mRNA during pre-mRNA splicing. In their examination of the spontaneous mutation rate and selective advantage of *HRAS* mutations in the paternal germline, Giannoulatou and co-workers [8] did not observe the c.35_36GC>TG mutation, whereas the c.35G>T, c.35_36GC>TT and c.35_36GC>TA p.Gly12Val mutations were observed. This is consistent with their hypothesis of selective advantage contributing to the observed abnormally high mutation rates in sperm, since the c.35_36GC>TG mutation would be selected against due to its deleterious effect on exon 2 inclusion.

**Fig 3:**
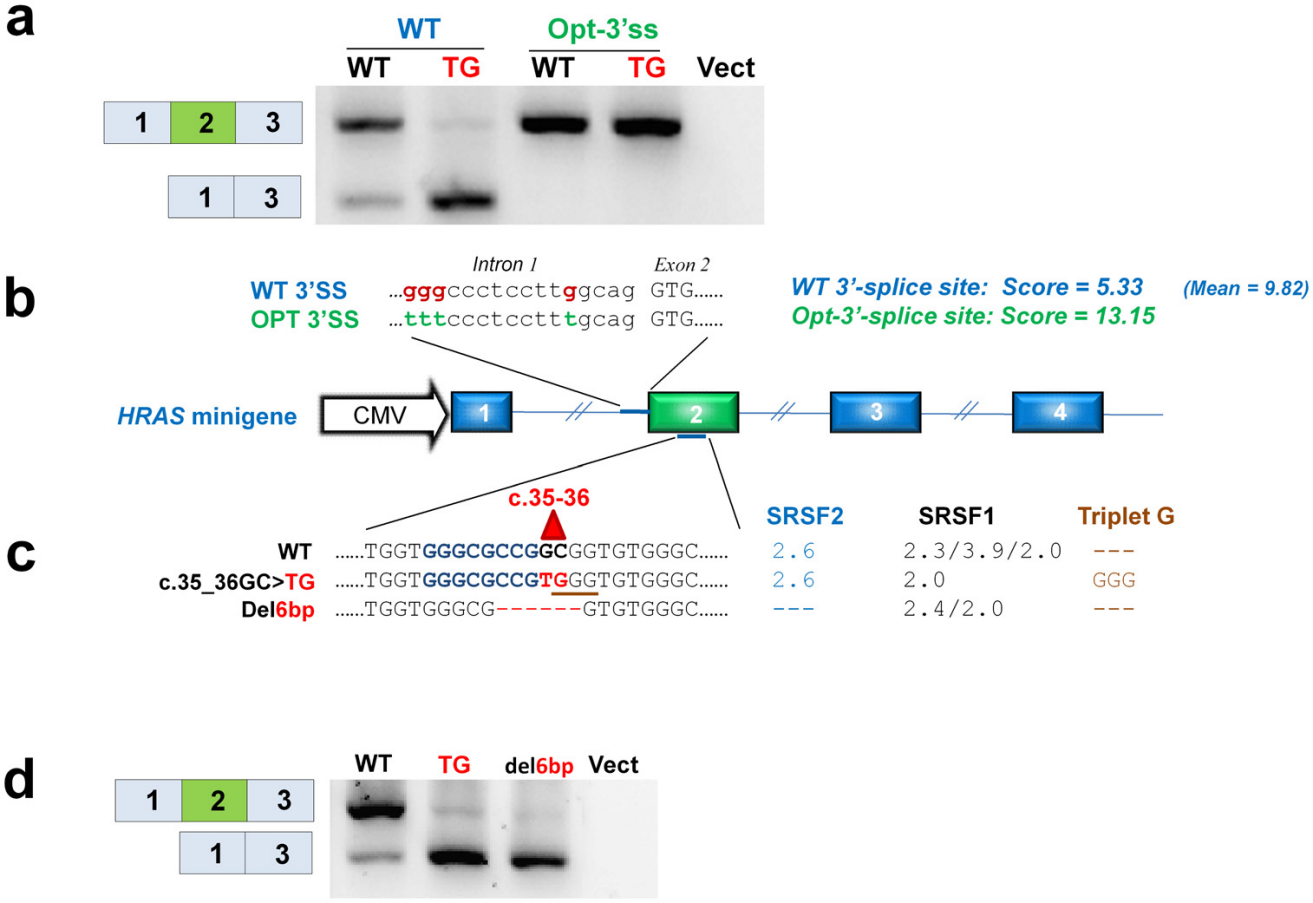
The weak 3’ splice site in *HRAS* exon 2 has a non-consensus G nucleotide and a GGG triplet in the polypyrimidine tract. (**a**) Representative results from HepG2 cells transfected with wild type and c.35_36GC>TG mutant minigenes holding either a weak wild type 3’ splice site or an optimized 3’-splice site. Introducing a strong 3’ splices site eliminates skipping of exon 2 indicating that the vulnerability of exon 2 is determined by the weak 3’ splice site. The lane labelled “Vect.” shows the results from a sample transfected with an empty p.cDNA3.1+ vector. (**b**) Displays the *HRAS* minigene construct. Sequences of the wild type and optimized 3’-splice sites are displayed. Scores based on MaxEnt calculations for wild type and optimized 3’-splice sites are listed. The mean score for all 3’-splice sites in the *HRAS* gene is shown. (**c**) Wild type, c.35_36GC>TG mutant and 6 bp deletion sequences are shown. The scores from ESE-finder and generation of an inhibitory GGG triplet are shown. (**d**) When a 6 bp deletion is introduced, exon 2 is completely skipped. The lane labelled “Vect.” shows the results from a sample transfected with an empty p.cDNA3.1+ vector.

Pre-mRNA splicing of constitutive exons with weakly defined splice sites is dependent on a delicate balance between exonic splicing enhancers (ESE) and exonic splicing silencers (ESS) [13,19]. ESEs bind positive splicing factors, typified by the serine/arginine-rich (SR) proteins [20]. In contrast, ESSs bind proteins from the heterogeneous nuclear ribonucleoprotein (hnRNP) family [21], which inhibits splicing. Consequently, a mutation which either disrupts/weakens a binding motif in an ESE or creates/strengthens an ESS can result in exon skipping if the exon is weakly defined. In silico analysis showed that *HRAS* exon 2 is weakly defined due to a weak 3’ splice site with a non-consensus G nucleotide at position -5 in intron 2 and a GGG triplet at positions -14 to -16 disrupting the polypyrimidine tract (Fig 3). A short polypyrimidine tract is difficult to recognize for the U2AF65 splicing factor, and furthermore GGG triplets can bind hnRNPF/H family proteins, which could compete with U2AF65 binding and thereby decrease 3’-splice site splicing efficiency [22]. We hypothesized that this weak 3’ splice site makes inclusion of *HRAS* exon 2 dependent on the binding of splicing regulatory proteins to ESEs and that this is disrupted by the c.35_36GC>TG mutation.

In order to make exon 2 recognition independent of the ESE/ESS balance we strengthened the weak 3’ splice site by replacement of the non-consensus G and the GGG triplet in the polypyrimidine tract with consensus T nucleotides (Fig 3). This improved splicing from the wild type *HRAS* minigene and abolished exon 2 skipping from the c.35_36GC>TG mutant. Interestingly, c.35_36GC>TG creates a known splicing inhibitory motif (G**TG**GGTG) (S3 Fig), which binds proteins from the hnRNPF/H family. There are several examples from other genes where single nucleotide substitutions have created or abolished this motif in a weak constitutive exon causing aberrant splicing and disease [22–27] (S3 Fig). This suggests that the c.35_36GC>TG mutation creates an hnRNPF/H binding ESS, which inhibits inclusion of exon 2. Consistent with this hypothesis, replacement in the *HRAS* minigene of the wild type sequence c.34-39 with known hnRNPF/H binding ESS motifs results in exon 2 skipping (S4 Fig). These motifs have previously been demonstrated to cause exon skipping in other genes [26,28]. Moreover, introduction of a single c.36C>G mutation, creating the hnRNPF/H (DGGGD) binding motif [29] also causes complete exon 2 skipping (S4 and S5 Figs) underscoring the importance of c.36G in disruption of splicing. However, both introduction of CC at position c.37-38 (S4 Fig) and deletion of nucleotides c.32-37 (Fig 3) in the wild type cause exon 2 skipping, indicating that a fundamental ESE is also present in this region of wild type *HRAS* exon 2.

**Fig 4:**
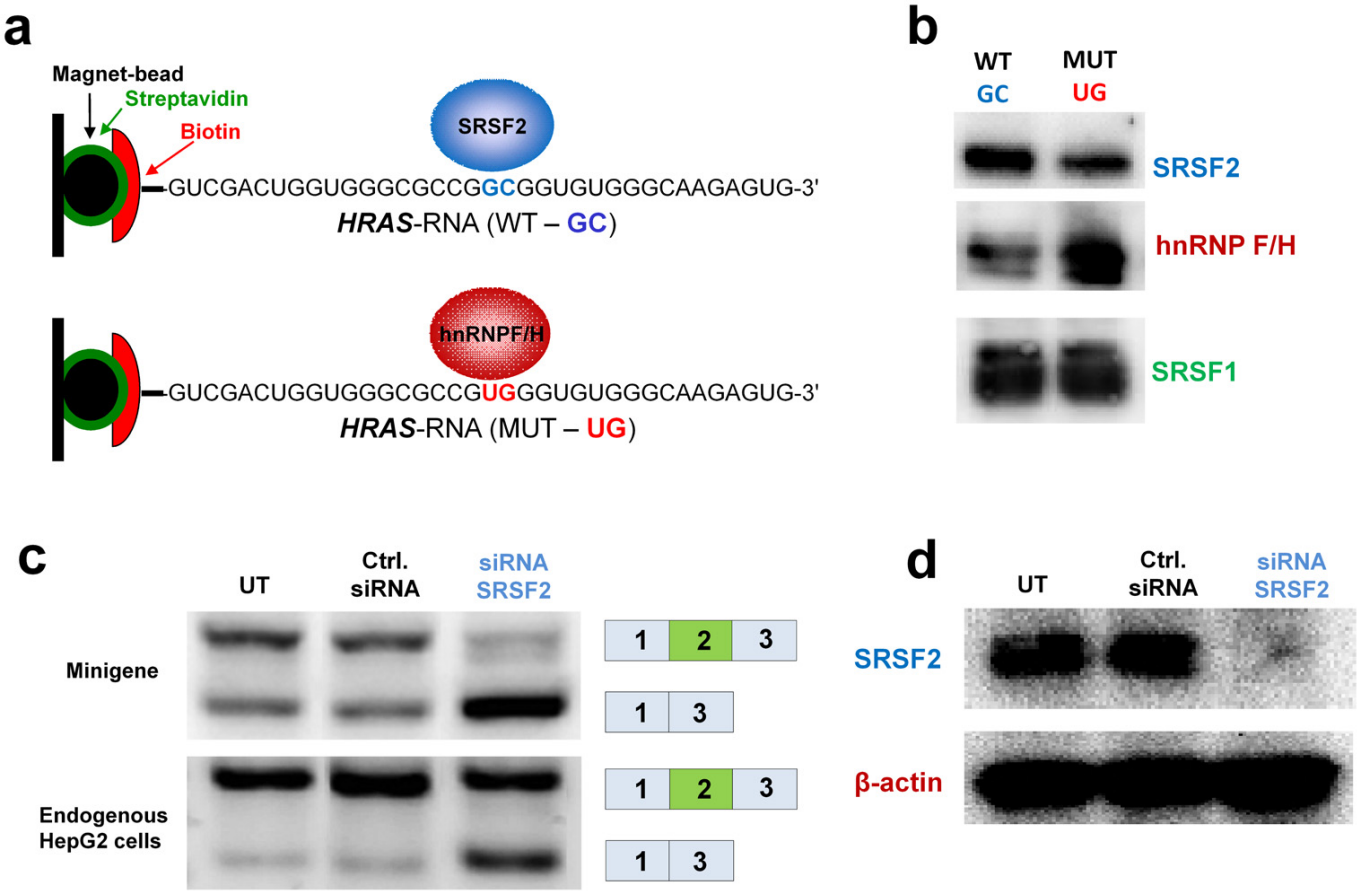
Binding analysis of SRSF2 and hnRNPF/H. (**a**) Biotinylated RNA oligonucleotides with either wild type or c.35_36TG *HRAS* sequence were incubated with HeLa nuclear extracts (NE). Biotinylated RNA oligonucleotides bind streptavidin coated beads allowing identification of protein-RNA motif interactions from NE. The beads are superparamagnetic and RNA binding proteins are purified when an external magnetic field is applied. (**b**) Western blot analysis shows that the c.35_36GC>TG mutation increases binding of hnRNPF/H proteins and decreases binding of SRSF2. (**c**) siRNA mediated knock down of SRSF2 causes exon 2 skipping both from the wild type *HRAS* minigene and endogenous *HRAS* in HepG2 cells. (**d**) Western blot analysis was used to confirm SRSF2 knock down.

We used two different splicing reporter minigenes [30,31] to demonstrate that this part of *HRAS* exon 2 harbors ESEs, which can drive splicing in other genomic contexts, and that exon inclusion is abolished by the c.35_36GC>TG mutation (S5 Fig). Interestingly, testing of the c.35G>T mutant sequence indicated that it results in more efficient splicing than the wild type sequence. This is consistent with the results from the *HRAS* minigene and indicates that this mutation may improve splicing. ESE finder analysis [32] also suggests that the region around c.35 harbors potential binding sites for the SRSF1 and SRSF2 splicing stimulatory proteins, which usually bind ESEs to stimulate splicing. The c.35_36GC>TG mutation directly abolishes SRSF1 motifs, but does not directly affect the SRSF2 motif, whereas a deletion of nucleotides c.32-37 disrupts both the SRSF1 and SRSF2 motifs (Fig 3). RNA affinity purification employing wild type and c.35_36GC>TG mutant RNA oligonucleotides combined with ITRAQ labeling followed by MS/MS analysis indicated that the c.35_36GC>TG mutation increases binding of hnRNP F/H proteins and decreases binding of SRSF2, but not SRSF1 and this could be demonstrated by western blot analysis (Fig 4). Binding of hnRNPF/H proteins may also disrupt binding of SRSF2 and other splicing stimulatory proteins to an overlapping ESE.

Since hnRNPF/H binding to GGG triplets in a pre-mRNA is cooperative and synergistic [29], mutations creating new GGG triplets in *HRAS* exon 2 are likely to inhibit splicing by acting in synergy with pre-existing GGG triplets, such as the flanking GGG triplets and the GGG triplet in the weak 3’-splice site.

Interestingly, expression of hnRNPF/H proteins is low in cardiomyocytes [33], suggesting that inclusion of c.35_36GC>TG mutant exon 2 could be high in the heart from our patient.

Taken together our data suggest that c.35_36GC>TG simultaneously disrupts an ESE and creates a strong hnRNPF/H binding ESS (S6 Fig). Consistent with this, siRNA mediated knock down of SRSF2 caused exon 2 skipping both from the wild type *HRAS* minigene and from endogenous *HRAS* in T24 and HepG2 cells (Figs 4 and S7), whereas SRSF1 knockdown had no effect on *HRAS* exon 2 inclusion (S8 Fig). This does of course not exclude that other splicing regulatory factors may also bind to the *HRAS* ESE and stimulate exon 2 inclusion.

**Fig 5:**
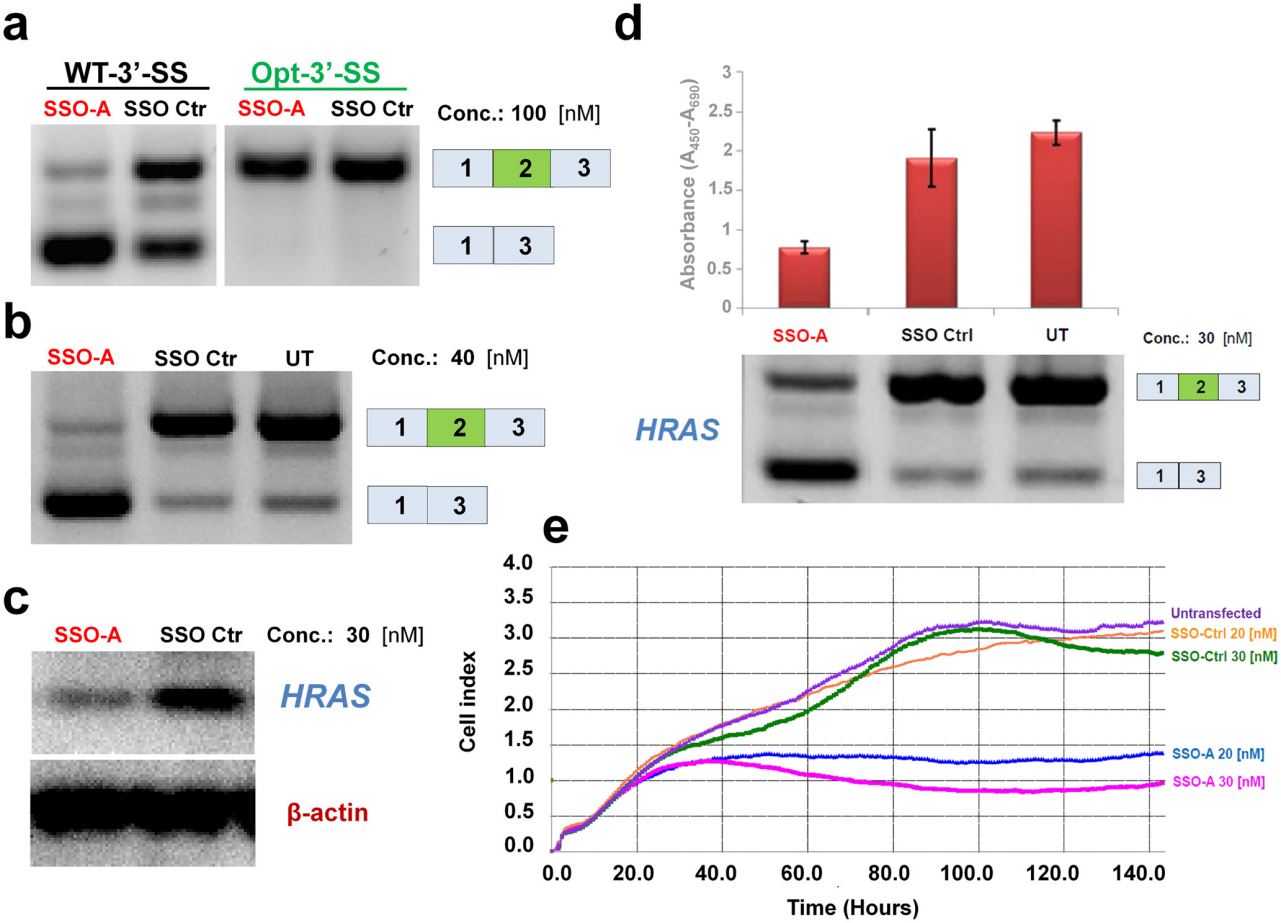
SSO-mediated skipping of *HRAS* exon 2. (**a**) T24 bladder cancer cells, which harbor the c.35G>T mutation, were transfected with *HRAS* minigenes with either a wild type or an optimized 3’ splice site and treated either with an SSO (SSO-A) that blocks access to the ESE or a scrambled control SSO. SSO-A treatment mediates exon 2 skipping from the wild type *HRAS* minigene, but this is alleviated when optimizing the 3’ splice site. (**b**) SSO-A treatment causes nearly complete skipping of endogenous *HRAS* exon 2 in T24 cells. (**c**) Western blot analysis confirmed reduced levels of HRAS protein following SSO-A treatment. (**d**) Quantification of cell viability after SSO-A treatment by WST-1 assay demonstrates that it decreases viability of T24 bladder cancer cells. (**e**) xCelligence real time monitoring of proliferation of T24 bladder cancer cells. Cells were treated with either SSO-A or control SSO at two concentrations (20 nM or 30 nM). When treated with SSO-A cell viability and growth is decreased.

To further substantiate that *HRAS* exon 2 skipping leads to inactivation of HRAS and that an ESE fundamental for *HRAS* exon 2 inclusion is present in the region harboring c.35G, we designed a splice switching oligonucleotide (SSO) that would block binding of splicing regulatory proteins to the ESE. Consistent with this proposed effect, the SSO caused exon 2 skipping from the wild type *HRAS* minigene. Interestingly, the effect of the SSO was alleviated when the 3’-splice site was strengthened in the minigene (Fig 5). This substantiates that vulnerability of exon 2 is determined by the weak 3’-splice site.

Next we demonstrated that the SSO causes exon 2 skipping from the endogenous *HRAS* gene in both T24 and HepG2 cells. This was reflected in reduced levels of HRAS protein and by decreased growth and proliferation (Fig 5). This indicates that *HRAS* mRNA with exon 2 skipped is either not translated due to the lack of the normal ATG start codon, or if a protein is produced from an alternative start codon the resulting protein is unstable. These data show that skipping of *HRAS* exon 2 leads to decreased growth and proliferation consistent with reduced *HRAS* activity. Moreover, they confirm that an important ESE is located around position c.35 and that SSO-mediated blocking of access to this ESE reduces exon 2 inclusion.

**Fig 6:**
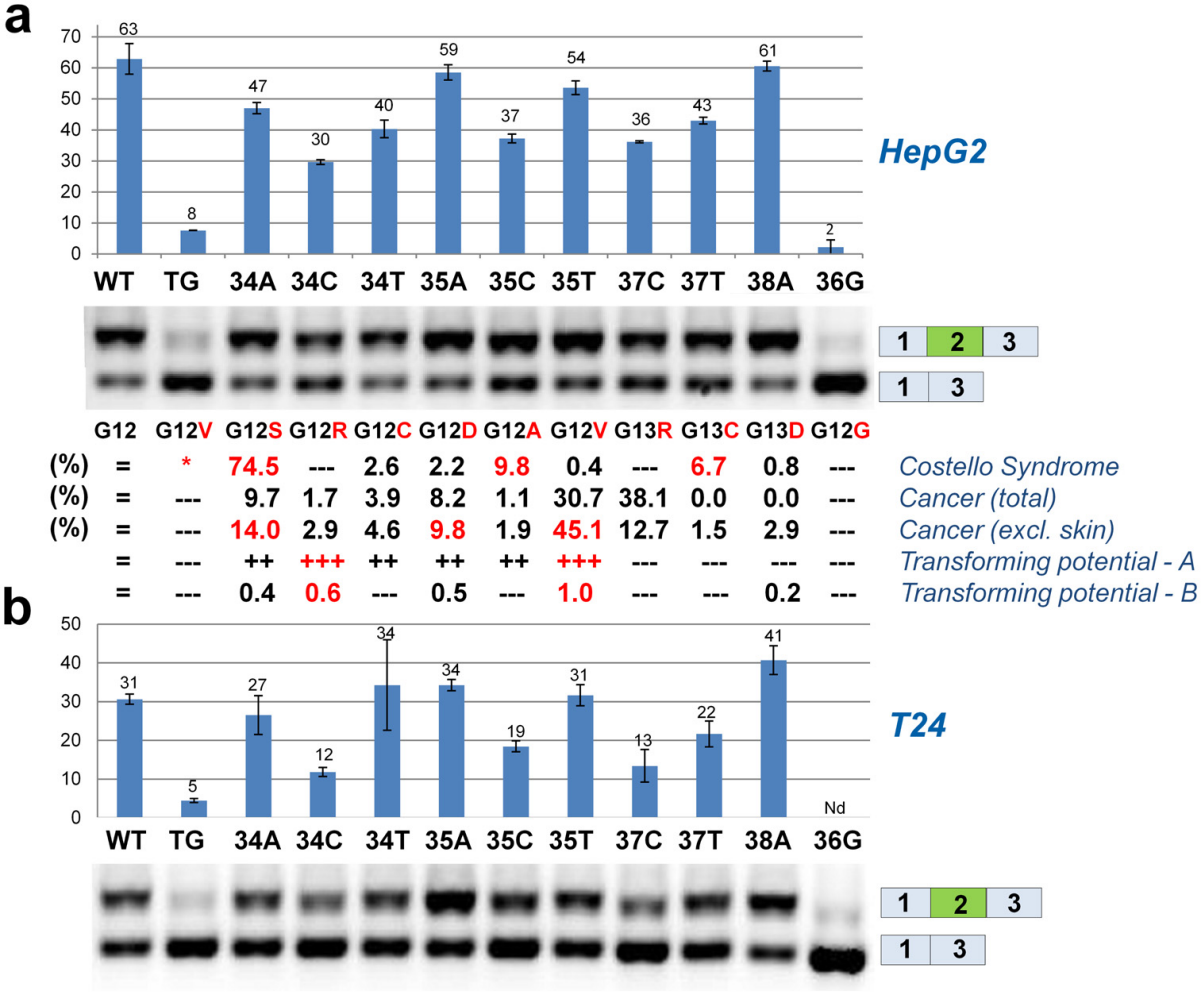
Mutations in codon 12 or 13 of *HRAS* exon 2 affect splicing differently. (**a**,**b**) HepG2 (*top*) or T24 (*bottom*) cells were transfected with *HRAS* minigenes harboring different sequence variants in positions c.34-38. The frequencies of the mutations in Costello syndrome according to Giannoulatou and co-workers [8] and in cancer according to Cosmic database are displayed. For cancer the numbers are displayed with skin cancers included or excluded due to the extremely high occurrence of the c.37G>C mutation in skin cancer. The original scoring of the transforming potential of the mutants in two studies are displayed—A is from Seeburg and co-workers [5]; B is from Fasano and co-workers [6]. Quantitative data for exon 2 inclusion (molar ratio) were obtained from triplicates of duplicate transfections using the Agilent 2100 Bioanalyzer. It is worth noting that there is a clear difference in the overall splicing efficiency between T24 cells and HepG2 cells, which is consistent with the reported low levels of hnRNPF in HepG2 cells [34].

Because recognition of ESEs by splicing stimulatory proteins is highly sequence specific, it is likely that other sequence variants in codon 12 and 13 may influence exon 2 inclusion and play a role in determining their phenotypic consequences. Consequently, we employed minigene transfection of T24 and HepG2 cells to test codon 12 and 13 mutations, which are known to cause CS [8] or cancer (Cosmic) for their effect on exon 2 inclusion (Fig 6). This showed that mutations like c.35G>C and c.37G>T, which are frequent in CS, but infrequent in cancer, have a relatively high level of exon 2 skipping, potentially attenuating their deleterious effect, whereas mutations like c.35G>A and c.35G>T, which are very frequent in cancers and rare in CS, have a high level of exon 2 inclusion (Fig 6).

In line with this, it is quite obvious that the c.35_36GC>TG mutation despite encoding the most severe mutant protein, p.Gly12Val, would most likely never be observed in cancer (c.35_36GC>TG is not present in the Cosmic database) due to the high level of exon 2 skipping and conversely, c.35G>T, which encodes an identical protein, is the second most frequent mutation in cancer and very rare in Costello syndrome due to the very efficient inclusion of exon 2.

The most frequent CS mutation, c.34G>A, has a very modest, nearly neutral, negative effect on exon 2 inclusion and its high occurrence in both CS and cancer is thus probably mainly due to a high mutation rate (due to CpG hypermutability) and a modest transforming potential of the encoded p.G12S protein.

## Discussion

We show that a particular mutation, c.35_36GC>TG, which encodes the prototypical oncogenic, constitutively active p.G12V HRAS protein, causes exon 2 skipping in an individual with CS with an attenuated clinical phenotype. This key finding demonstrates, *in vivo*, in a patient that exon 2 skipping leads limited production of the constitutively active oncogenic HRAS, thereby attenuating clinical symptoms. Simultaneously, this points to a previously unrecognized “Achilles heel” of the *HRAS* gene, namely that exon 2 is weakly defined due to a suboptimal 3’splice site. It’s inclusion in the mRNA is therefore dependent on binding of splicing stimulatory proteins, like SRSF2 to ESEs, and that binding of splicing inhibitory proteins, like hnRNPF/H to ESSs is avoided. Thus *HRAS* exon 2 inclusion can be affected by mutations altering the balance between ESEs and ESSs and this could in turn also result in cell type specific differences in splicing efficiency dependent on the relative levels/activities of splicing regulatory proteins, like SRSF2 or hnRNPF/H in the relevant tissue. Consistent with this, we show that mutations in codon 12 and 13 (c.34-39) impact exon 2 inclusion differently, and that *HRAS* exon 2 splicing efficiency is different (e.g. T24 and HepG2 cells). Thus, our results illustrate that the oncogenic effect of different mutations in *HRAS* may be determined also by their effect on exon 2 splicing efficiency. This adds an additional layer to the complex interpretation of the molecular consequences of mutations in *HRAS* exon 2. We posit that a delicate balance exists between the mutability of the different nucleotides, the resulting efficiency of exon 2 inclusion, and the oncogenic effect of the encoded mutant protein. We postulate that the observed frequencies of the various mutations in codon 12 and 13 in CS and cancers are a result of this balance.

This has clear implications for our understanding of the correlation between genotype and phenotype in diseases caused by *HRAS* mutations and highlights the general importance of the “splicing code” [13], by providing a striking example on how exonic mutations, like c.35_36GC>TG, can affect splicing and have dramatically different effects than those predicted based solely on the genetic code.

Finally, we show that this previously unknown weakness of the *HRAS* gene points to a new mechanism for knocking out oncogenic HRAS by employing SSOs that block binding of the required splicing regulatory factors, resulting in exon 2 skipping and decreased growth of cancer cells. SSOs targeting *HRAS* exon 2 splicing may represent a new therapeutic approach either when used alone or in combination with other therapies. In contrast to traditional drugs, SSOs are highly specific for a single gene and there are currently promising clinical trials employing SSOs for treating human disease. SSOs that are able to inhibit tumorigenesis *in vivo* by altering splicing of genes, like *Bcl-X*, *STAT3* and *MDM4* have been reported [35–37]. A particularly appealing characteristic of SSOs is that delivery of several different SSOs targeting different cancer genes, like those mentioned above, could be performed simultaneously using the same delivery method. In this way multiple oncogenic mechanisms could be targeted in a single approach. Our data suggest that SSO-based therapy targeting *HRAS* could also be included in such a future strategy.

## Materials and Methods

### Ethics statement

Written informed consent was obtained from all participants and the study was approved by the Institutional Review Board at the University of Utah (IRB#00013747).

### Clinical review

This 12-year-old boy had a history of hypertrophic cardiomyopathy status post septal myomectomy at 11 months of age. An echocardiogram at 12 years showed only mild septal hypertrophy with trace aortic insufficiency. A Nissen fundoplication and gastrostomy tube (GT) placement were performed at 2 months of age due to swallowing dysfunction and aspiration. The GT was used intermittently for 10 years but subsequently removed. He had one generalized seizure at 1 year without recurrence. A brain MRI at 17 months showed mild enlargement of the lateral and third ventricles with a mild Chiari I malformation. He received growth hormone injections starting at 9 years of age due to growth hormone deficiency. At 12 years, growth parameters were as follows: height = 142 cm (10^th^ centile), weight = 32 kg (5^th^ centile), and head circumference = 55.5 cm (75^th^ centile). Other clinical features included ptosis, telecanthus, posteriorly rotated ears, deviated nasal septum, dental crowding, retrognathia, slightly large appearing hands without significant skin redundancy/deep creases or ulnar deviation, pes planus, an asymmetric anterior chest wall deformity, hyperflexibility, and mild kyphosis. He had one large nevus on his leg but otherwise did not have any additional dermatologic abnormalities and his hair appeared normal. There was no history of malignancies. He had mild developmental delay with good verbal skills and a full scale IQ of 76. He required resource classes for approximately 40% of his classes but was in the mainstream educational system for the remaining classes with some modification.

### *HRAS* minigenes

Genomic DNA was used for PCR amplification of a fragment of the human *HRAS* gene (NC_000011.9) encompassing exons 1–4 using Platinium Pfx DNA Polymerase supplemented with enhancer solution (Invitrogen) and primers HRAS1sNheI: 5’-GGCCCCGCTAGCAGTCGCGCCTGTGAA-3’ and HRAS1asXhoI: 5’-GTGAAGGACTCGAGTGACGTGCCCAT-3’. The amplified fragment was digested with *NheI* and *XhoI* and cloned into the polylinker of pcDNA.3.1+ (Invitrogen). Mutations were introduced by site-directed mutagenesis using standard methods either by the authors or by GeneScript Inc. (GenScript, Piscataway, NJ, USA). All plasmids were sequenced by GATC Biotech AG (Germany) in order to exclude any PCR derived errors.

### RHCglo and pSXN splicing reporters

*HRAS* exon 2 and variant double stranded DNA oligonucleotides corresponding to c.13_47 of *HRAS* exon 2 were inserted into the alternatively spliced second exon in the RHCglo splicing reporter minigene [31]. To generate pSXN constructs [30] we used sense and antisense oligonucleotides with desired sequences. The integrity of all constructs was confirmed by sequencing.

### Transient transfections of HepG2 and T24 cells and splicing analysis

T24 human urinary bladder cancer cells were obtained from Coriell Institute (https://catalog.coriell.org/). HepG2 human hepatocellular carcinoma cells were obtained from American Type Culture Collection (ATCC) (http://www.lgcstandards-atcc.org/en.aspx). HepG2 or T24 cells were grown under standard conditions using 10% RPMI (Lonza RPMI 1640 added 10% FCS, glutamine (100x) and pen/strep (1000 U/ml)) or 5% RPMI (Lonza RPMI 1640 added 5%FCS, glutamine (100x) and pen/strep (1000 U/ml)), respectively. Twenty-four hours before transfection the cells were seeded 9.6 cm^2^ 6-well plates (Nunc) at a density of 1.7×10^5^ (HepG2) or 1.2×10^5^ (T24) in 2 ml 5% or 10% RPMI (25% confluence) and grown O.N. to a density of 50% confluence on the day of transfection. Cells were transfected with a total DNA amount of 800 ng per well using X-tremeGene 9 DNA Transfection Reagent (Roche). Cells were transfected with 600 ng of plasmid DNA of interest and co-transfected with 200 ng MCAD 362T plasmid [19] as positive control. Forty-eight hours after transfection cells were harvest for RNA using Isol-RNa Lysis Reagent (AH Diagnostic) and RNA isolated using phenol-chloroform extraction. cDNA synthesis was performed using Superscript VILO cDNA Synthesis Kit (Invitrogen). Splicing analysis was carried out by PCR amplification and agarose gel electrophoresis. For *HRAS* constructs we used a specific primer T7-EXT: 5’- ATTAATACGACTCACTATAGGG-3’ and a primer spanning the exon 3-exon 4 junction of the *HRAS* gene (RasEx4Ex3: 5’-CGTTTGATCTGCTCCTGTAC-3’). For the RHCglo constructs we used primers RSV5U: 5′-CATTCACCACATTGGTGTGC-3′ and TNIE4: 5′-AGGTGCTGCCGCCGGGCGGTGGCTG-3′. For the pSXN construct we used primers pSXN12s2: 5'-AAGGTGAACGTGGATGAAGTTGGTGGTG-3' and pSXN13as: 5'-CCCACGTGCAGCCTTTGACCTAGTA-3'. All transfections were performed in triplicates.

### RNA affinity purification of nuclear proteins

Affinity purification of RNA binding proteins was performed with 3’-biotin coupled RNA oligonucleotides (DNA Technology, Denmark) as previously described [19]. The sequences of the RNA oligonucleotides were: HRAS-wt (5′-GUCGACUGGUGGGCGCCGGCGGUGUGGGCAAGAGUG-3′) and HRAS-mut (5′-GUCGACUGGUGGGCGCCGUGGGUGUGGGCAAGAGUG-3′) corresponding to position c.17_52 of *HRAS* mRNA. For each purification 100 pmol of RNA oligonucleotide was coupled to 100 μl of streptavidin-coupled magnetic beads (Invitrogen) and incubated with HeLa nuclear extract (Cilbiotech S.A., Belgium). Eluted proteins were analyzed by western blotting using a monoclonal anti-SRSF2 antibody (sc-041550 from Millipore) or a polyclonal anti-hnRNPF/H (sc-15387 from Santa Cruz).

### Splice shifting oligonucleotide design

SSOs were phosphorothioate oligonucleotides with 2'-O-methyl modification on each sugar moiety (DNA-technology, Denmark). HRAS-SSO-A: 5’-CGCACUCUUGCCCACACCGCCGGCG-3’ (Nucleotides corresponding to pos. c.51_30) and control SSO: 5’-GCUCAAUAUGCUACUGCCAUGCUUG-3'.

### Reverse transfection of HepG2 or T24 cells with SSOs

Approximately 3×10^5^ HepG2 or T24 cells were reverse transfected with 50 pmol (20 nM), 75 pmol (30 nM), 100 pmol (40 nM) or 250 pmol (100 nM) of SSOs using Lipofectamine RNAiMAX transfection reagent (Invitrogen). Forty-eight hours after transfection cells were harvest for RNA using Isol-RNa Lysis Reagent (AH Diagnostics, Denmark) and RNA isolated using phenol-chloroform extraction or analyzed by the WST-1 viability assay. cDNA synthesis was performed using Superscript VILO cDNA Synthesis Kit (Invitrogen). For exogenous splicing analysis cells were transfected with minigene constructs 24 hours after SSO treatment. Splicing analysis of endogenous *HRAS* transcripts were performed by PCR with primers located in exon 1 (HRAS1sNheIS: 5’-GGCCCCGCTAGCAGTCGCGCCTGTGAA-3’) and a primer spanning the exon 3-exon 4 junction of the *HRAS* gene (RasEx4Ex3AS: 5’-CGTTTGATCTGCTCCTGTAC-3’). For exogenous splicing analysis we used primers T7-EXT: 5’- ATTAATACGACTCACTATAGGG-3’ and RasEx4Ex3AS: 5’-CGTTTGATCTGCTCCTGTAC-3’. All experiments were performed at least in triplicate. To check the effect of SSO treatment on HRAS protein levels, protein was extracted for SDS-PAGE and western blotting using a mouse polyclonal anti-HRAS antibody (SAB1405964 from Sigma-Aldrich) or an rabbit anti-beta actin antibody (ab8229 from AbCam).

### Determination of cell viability

Cell viability was determined by the WST-1 viability assay in 96 well plates following the manufacturer’s instructions (Roche). Approximately 3×10^5^ T24 cells/well were reverse transfected with 30nM of SSOs using Lipofectamine RNAiMAX transfection reagent (Invitrogen) and incubated for 72h. Absorbance was measured on a VERSAmax tunable microplate reader (Molecular devices) at 3, 4 and 5 hours after addition of the WST-1 reagent. Non-treated cells, cells treated only with Lipofectamine RNAiMAX transfection reagent (Invitrogen) and a non-targeting scrambled SSO served as controls. All WST-1 viability assays were performed at least in triplicate.

### siRNA mediated knock down

Knockdown of SRSF1 or SRSF2 was performed using 100 pmol siRNA SMARTpools (Thermo Scientific) and scrambled control. For endogenous knock down T24 and HepG2 cells were grown to a density of 60–80% confluence when treated with siRNA using Lipofectamine RNAiMAX transfection reagent according to manufacturer’s instructions. Forty-eight hours after transfection cells were harvest for RNA using Isol-RNa Lysis Reagent (AH Diagnostics) and RNA isolated using phenol-chloroform extraction or protein was extracted for SDS-PAGE and Western blotting. For exogenous knock down cells were transfected with minigene constructs 24 hours after siRNA treatment.

### Xcelligence assay

Approximately 9000 T24 cells/well were reverse transfected with 20 nM or 30 nM of SSO-A or control SSO using Lipofectamine RNAiMAX transfection reagent (Invitrogen) and cell index was continuously monitored for 140 h. Real-time proliferation analysis was conducted using E plates (Roche, Basel, Switzerland) and xCELLigence kinetic Systems (ACEA Biosciences, San Diego, CA). The xCELLigence software (RTCA 1.2) was used to collect impedance measurements (reported as Cell Index) every 10 min for up to 72 hours.

### Real-time qPCR

Real-time (RT) qPCR and analysis were performed using a LightCycler with software version 1.5.1.62 (Roche). The RT qPCR master mix was prepared using the FastStart Essential DNA Green Master (Roche). For quantification of total *HRAS* we used primers HRASEX1S: 5’-CAGTCGCGCCTGTGAACGGTGG-3’ and HRASEX3-2AS: 5’-CCTGCTTCCGGTAGGAATCCTCTATAGTGGG-3’. For exon 2 skipping analysis we used primers HRASEX1-3S: 5’-CGCGCCTGTGAACGGATTCC-3’ and HRASEX4-Ex3-QPCR2AS: 5’-CACCCGTTTGATCTGCTCCTGTACT-3’.

## Supporting Information

S1 Fig Sequence analysis of *HRAS* exon 2 in genomic DNA from different tissues. Four different human tissues (blood, cheek swab, hair and urine) from index individual with Costello syndrome were sequenced for *HRAS* exon 2. *HRAS* sequencing identified a c.35_36GC>TG (p.G12V) mutation in all tissues sampled without evidence of mosaicism.
(TIF)

S2 Fig Analysis of *HRAS* exon 2 skipping in cDNA from different human tissues. (**a**) cDNA from eight different human tissues was tested for *HRAS* exon 2 skipping. Each tissue displayed a low level of *HRAS* exon 2 skipping, which varied between tissues. Primers located in exon 1 (HRAS1sNheIS) and spanning the exon 4–3 junction (RasEx4Ex3AS) allows simultaneous detection of products with and without exon 2 included. A primer set specific for the exon 2 skipped (HRASEX1-3S and RasEx4Ex3AS) was used to amplify only the exon skipped product. RPL13A was amplified as a control. H; Heart, B; Brain, PI; Placenta, Lu; Lung, Li; Liver, SM; Skeletal muscle, Ki; Kidney, Pa; Pancreas. (**b**) QPCR analysis for total *HRAS* mRNA (*top*) using primers HRASEX1S and HRASEX3-2AS or exon 2 skipping (*bottom*) using primers HRASEX1-3S and HRASEX4-Ex3-QPCR2AS was performed in samples from 20 different human tissues and normalized to *RPL13A* (Nearly identical data were obtained when we used the *TBP* gene for normalization instead).
(TIF)

S3 Fig Analysis of disease-causing splicing regulatory motifs having similar architecture as the one observed in *HRAS* exon 2. The Fig shows alignment of the sequence surrounding the c.35_36GC>TG mutation with 11 nucleotides flanking sequence to each side. The conserved splicing silencer motif (GTGGGTG) is boxed in red. Relevant sequences from the genes are listed with disease-causing variations marked in red. The genes listed are *CHRNA1*, *ACADSB*, *HEXB*, *INSR* and *FVIII*. In intron 3 of *CHRNA1* a disease-causing mutation disrupts a GGG triplet located in the polypyrimidine tract of the 3’ splice site flanking the non-functional alternative exon P3A, thereby excluding binding of hnRNPF/H proteins and causing aberrant exon inclusion [25]. Disease causing mutations in exon 10 of *ACADSB* and *HEXB* exon 12, which also create this GTGGGTG motif, result in exon skipping and disease [22,23,26]. The core GTGGGTG motif created by the c.35-36 mutation is also found in the insulin receptor gene (*INSR*) where hnRNPF binding to this element in intron 10 is involved in regulating alternative splicing of exon 11 [27]. Additionally, a C>T substitution in exon 19 of *FVIII* also creates this motif TGGTGGGTGG and causes exon skipping [24]. This suggests that the c.35_36GC>TG mutation creates an hnRNPF/H binding ESS, which inhibits inclusion of exon 2. Since hnRNPF/H binding to GGG triplets in a pre-mRNA is cooperative and synergistic [29], it is likely, that hnRNPF/H binding to the created ESS is synergistic with other flanking GGG triplets (underscored) and that this also facilitates binding of hnRNPF/H to the GGG triplet in the weak polypyrimidine tract and that this contributes to the exon skipping effect. *CHRNA1*: cholinergic receptor, nicotinic, alpha 1; *ACADSB*: short/branched chain acyl-CoA dehydrogenase; *HEXB*: hexosaminidase B (beta polypeptide); *INSR*: insulin receptor; FVIII: Factor (F) VIII.
(TIF)

S4 Fig Demonstrating that both creation of hnRNPF/H binding motifs and disruption of the ESE motif in *HRAS* exon 2 causes skipping. (**a**) Displays the *HRAS* minigene construct and replacement of the wild type sequence c.34-39 with known hnRNPF/H binding ESS motifs (CAGGGT or TAGGGA). A single c.36C>G mutation was also introduced to create the hnRNPF/H (DGGGD) binding motif [29]. A dinucleotide c.37_38GG>CC mutation was introduced to disrupt the ESE motif. (**b**) Representative results from cells transfected with wild type and mutant minigenes. Agarose gel electrophoresis reveals exon 2 skipping both when an hnRNPF/H motif is introduced and when the ESE is disrupted by introduction of c.37_38GG>CC.
(TIF)

S5 Fig Testing *HRAS* exon 2 sequences in two splicing reporter minigenes. (**a**) Schematic overview of the RHCglo splicing reporter and construct used in this study, harboring either *HRAS* wild type sequence or c.35G>T, c.35G>C, c.35_36GC>TG or c.35_36GC>TT mutant sequences (top). The second exon in the RHCglo splicing reporter is immediately flanked upstream and downstream by the last and first 91 and 73 nucleotides of human β-globin intron 1, respectively. The distal upstream segment of intron 1 contains introns 1 and 3 of chicken skeletal troponin I (sTNI), and the distal downstream region of intron 2 contains the last 364 nucleotides of sTNI intron 3. Inclusion of the alternatively spliced second exon is critically dependent on the balance between ESEs and ESSs in the inserted sequence. PCR analysis of splicing of RHCglo-*HRAS* constructs in HepG2 cells (bottom). (**b**) Schematic overview of the pSXN splicing reporter and construct used in this study, holding either *HRAS* wild type sequence c.35_36GC>TG or c.36C>T or c.36C>G mutated sequences (top). The pSXN reporter contains an artificial small (34 bp) exon positioned between β-actin exon 1 and exon 2 as well as downstream exon 18 from *cTNT*. The natural sequence of β-actin intron 1 is inserted on both sides of the middle exon. The reporter contains flanking *Sall* and *BamHI* restriction sites for cloning. PCR analysis of splicing of pSXN-*HRAS* minigenes in HepG2 cells (bottom).
(TIF)

S6 Fig The c.35_36GC>TG mutation identified in the index individual with Costello syndrome disrupts an SRSF2 binding ESE and creates an hnRNPF/H binding ESS within exon 2. (**a**) Splicing of *HRAS* exon 2 depends on SR-proteins like SRSF2 to be recognized by the spliceosome due to a suboptimal 3’splice site. Binding of SRSF2 within exon 2 promotes its inclusion in the *HRAS* mRNA. (**b**) The c.35_36GC>TG mutation in exon 2 disrupts the ESE and creates an ESS, which binds hnRNPF/H and thereby excludes inclusion of exon 2 in the mRNA, since it cannot be recognized by the spliceosome.
(TIF)

S7 Fig Endogenous SRSF2 knock down in T24 cells. (**a**) PCR analysis of *HRAS* exon 2 skipping reveals skipping in the context of SRSF2 knock down. (**b**) Western blot analysis confirmed reduced levels of SRSF2 protein after knock down. β-actin was used as a control.
(TIF)

S8 Fig Endogenous SRSF1 knock down in T24 cells. (**a**) PCR analysis reveals no effect on splicing of *HRAS* exon 2 after SRSF1 knock down. (**b**) Western blot analysis confirmed reduced levels of SRSF1 protein after knock down.
(TIF)
