# Supplementary figures and images for: The Splicing Efficiency of Activating HRAS Mutations Can Determine Costello Syndrome Phenotype and Frequency in Cancer
Source: PLoS Genet. 2016 May 19;12(5):e1006039. doi: 10.1371/journal.pgen.1006039 (PMC4873146; doi:10.1371/journal.pgen.1006039)

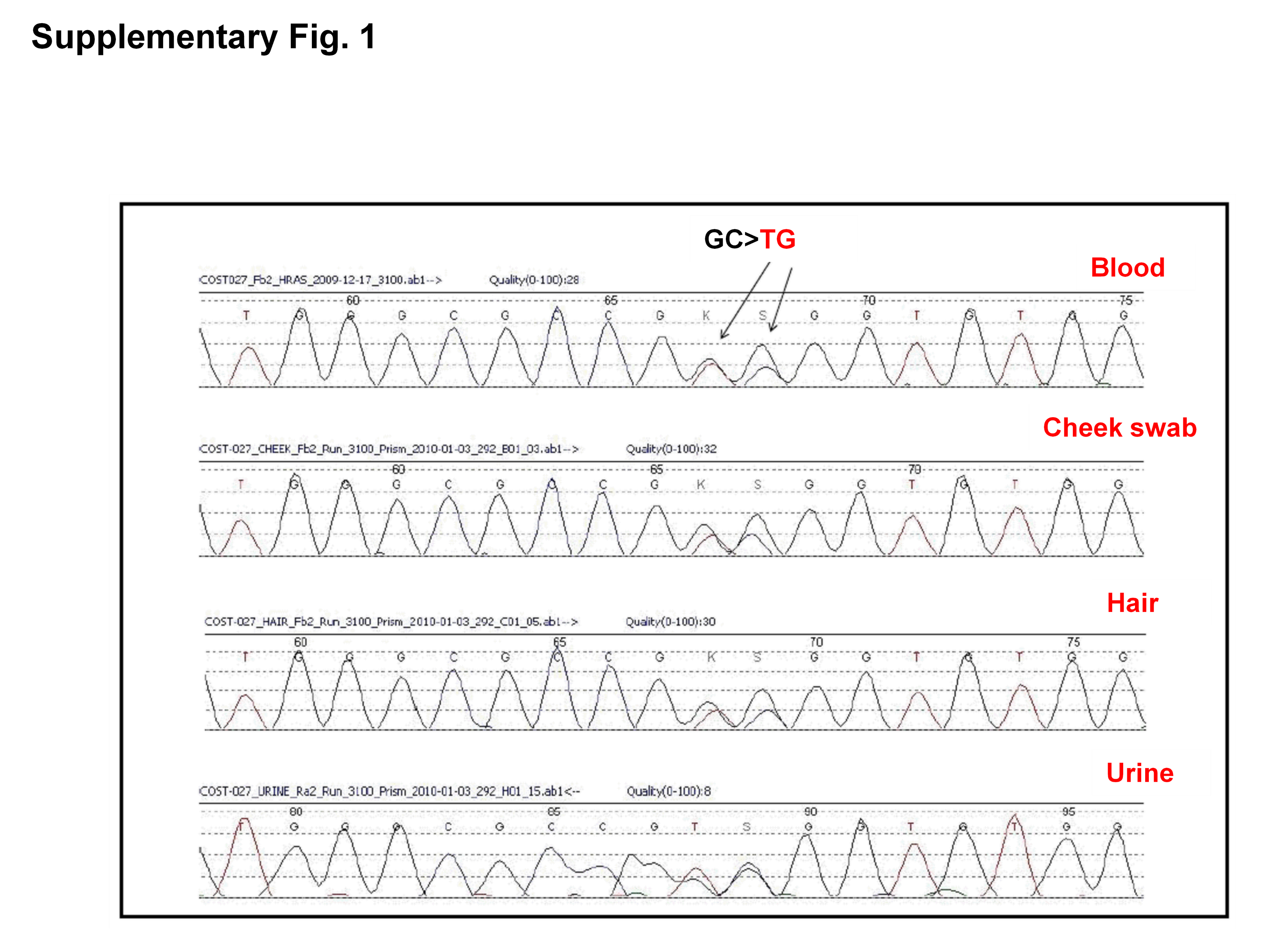

Supplement: S1 Fig — Four different human tissues (blood, cheek swab, hair and urine) from index individual with Costello syndrome were sequenced for HRAS exon 2. HRAS sequencing identified a c.35_36GC>TG (p.G12V) mutation in all tissues sampled without evidence of mosaicism. (TIF) [file pgen.1006039.s001.tif]

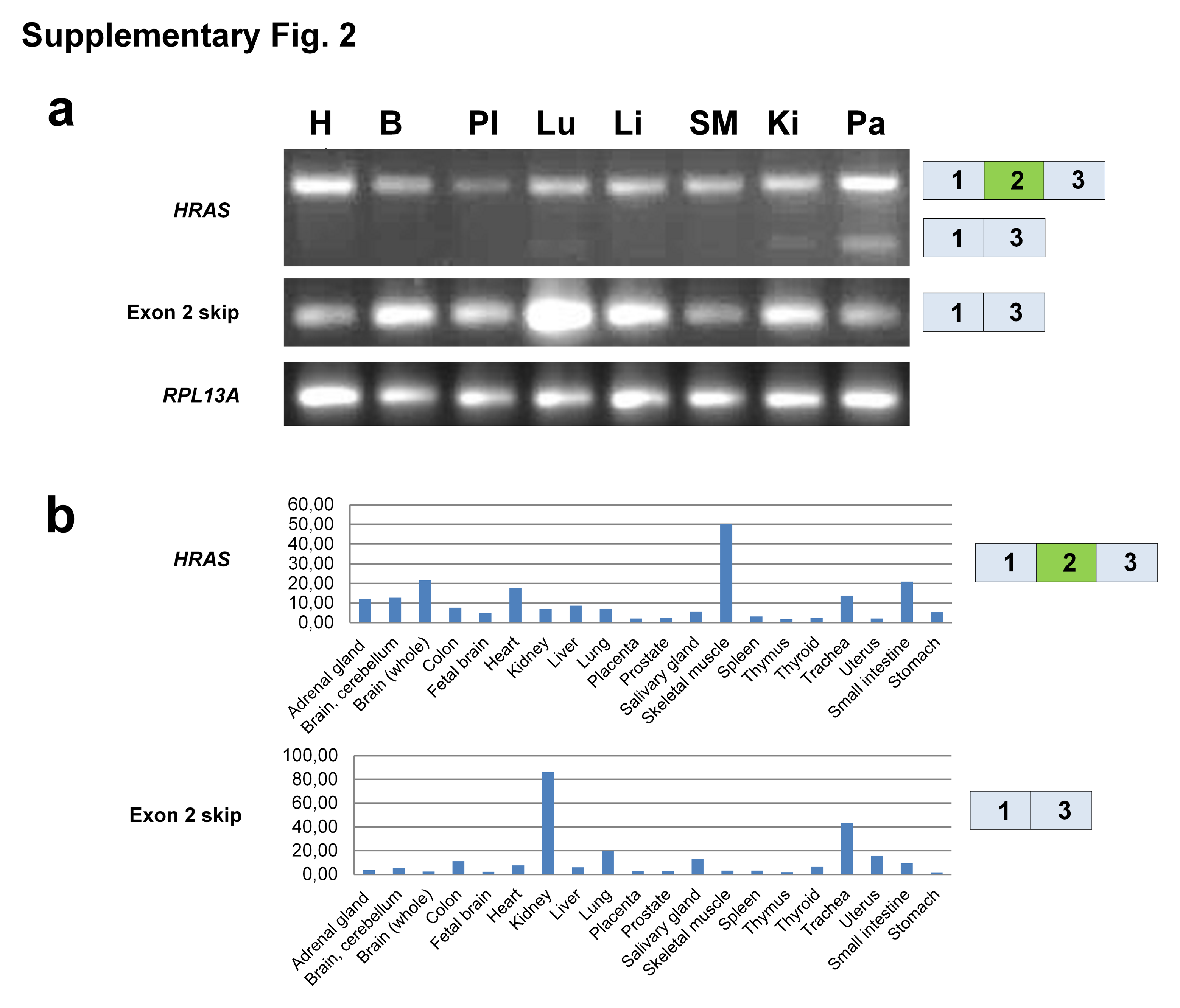

Supplement: S2 Fig — (a) cDNA from eight different human tissues was tested for HRAS exon 2 skipping. Each tissue displayed a low level of HRAS exon 2 skipping, which varied between tissues. Primers located in exon 1 (HRAS1sNheIS) and spanning the exon 4–3 junction (RasEx4Ex3AS) allows simultaneous detection of products with and without exon 2 included. A primer set specific for the exon 2 skipped (HRASEX1-3S and RasEx4Ex3AS) was used to amplify only the exon skipped product. RPL13A was amplified as a control. H; Heart, B; Brain, PI; Placenta, Lu; Lung, Li; Liver, SM; Skeletal muscle, Ki; Kidney, Pa; Pancreas. (b) QPCR analysis for total HRAS mRNA (top) using primers HRASEX1S and HRASEX3-2AS or exon 2 skipping (bottom) using primers HRASEX1-3S and HRASEX4-Ex3-QPCR2AS was performed in samples from 20 different human tissues and normalized to RPL13A (Nearly identical data were obtained when we used the TBP gene for normalization instead). (TIF) [file pgen.1006039.s002.tif]

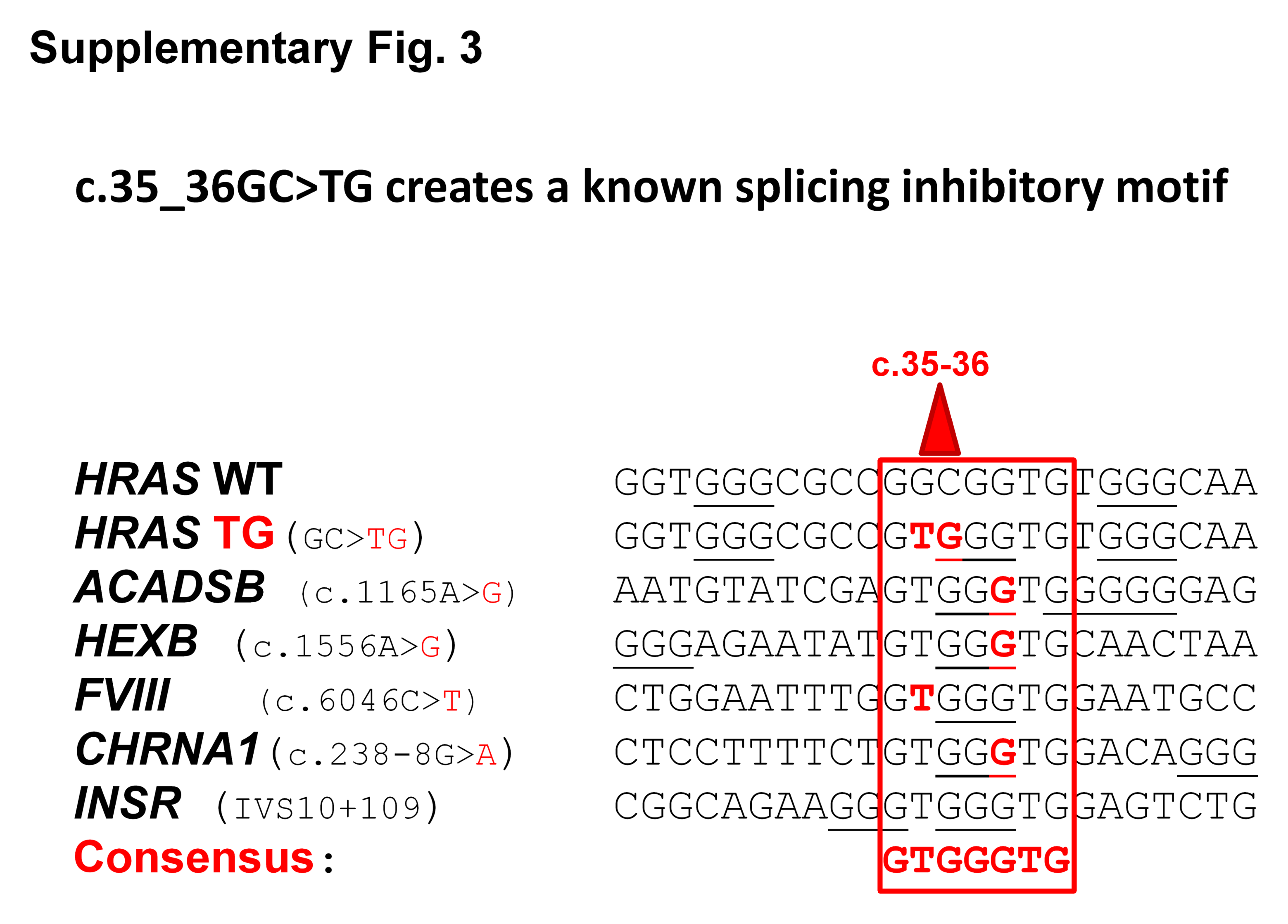

Supplement: S3 Fig — The Fig shows alignment of the sequence surrounding the c.35_36GC>TG mutation with 11 nucleotides flanking sequence to each side. The conserved splicing silencer motif (GTGGGTG) is boxed in red. Relevant sequences from the genes are listed with disease-causing variations marked in red. The genes listed are CHRNA1, ACADSB, HEXB, INSR and FVIII. In intron 3 of CHRNA1 a disease-causing mutation disrupts a GGG triplet located in the polypyrimidine tract of the 3’ splice site flanking the non-functional alternative exon P3A, thereby excluding binding of hnRNPF/H proteins and causing aberrant exon inclusion [25]. Disease causing mutations in exon 10 of ACADSB and HEXB exon 12, which also create this GTGGGTG motif, result in exon skipping and disease [22,23,26]. The core GTGGGTG motif created by the c.35-36 mutation is also found in the insulin receptor gene (INSR) where hnRNPF binding to this element in intron 10 is involved in regulating alternative splicing of exon 11 [27]. Additionally, a C>T substitution in exon 19 of FVIII also creates this motif TGGTGGGTGG and causes exon skipping [24]. This suggests that the c.35_36GC>TG mutation creates an hnRNPF/H binding ESS, which inhibits inclusion of exon 2. Since hnRNPF/H binding to GGG triplets in a pre-mRNA is cooperative and synergistic [29], it is likely, that hnRNPF/H binding to the created ESS is synergistic with other flanking GGG triplets (underscored) and that this also facilitates binding of hnRNPF/H to the GGG triplet in the weak polypyrimidine tract and that this contributes to the exon skipping effect. CHRNA1: cholinergic receptor, nicotinic, alpha 1; ACADSB: short/branched chain acyl-CoA dehydrogenase; HEXB: hexosaminidase B (beta polypeptide); INSR: insulin receptor; FVIII: Factor (F) VIII. (TIF) [file pgen.1006039.s003.tif]

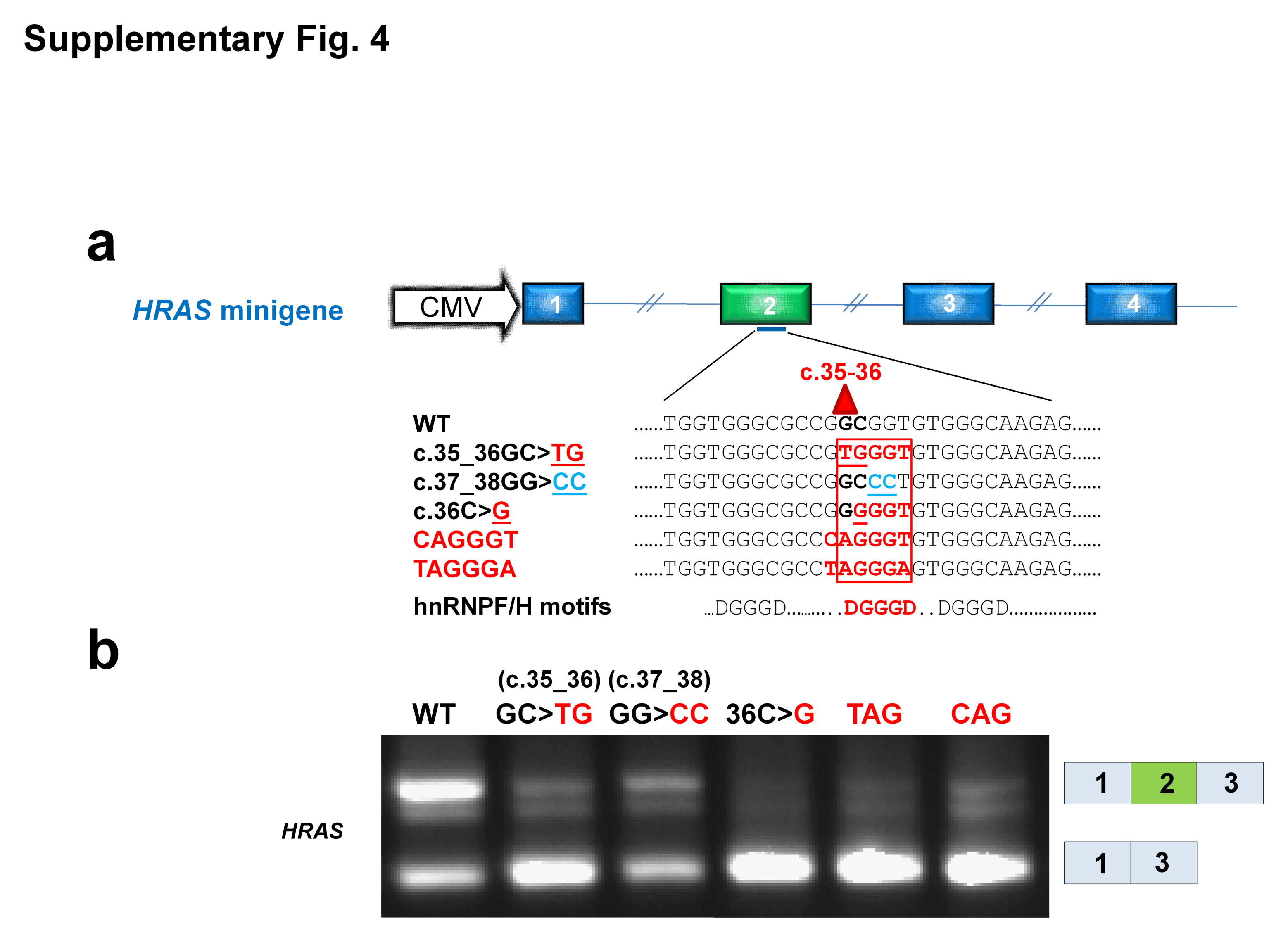

Supplement: S4 Fig — (a) Displays the HRAS minigene construct and replacement of the wild type sequence c.34-39 with known hnRNPF/H binding ESS motifs (CAGGGT or TAGGGA). A single c.36C>G mutation was also introduced to create the hnRNPF/H (DGGGD) binding motif [29]. A dinucleotide c.37_38GG>CC mutation was introduced to disrupt the ESE motif. (b) Representative results from cells transfected with wild type and mutant minigenes. Agarose gel electrophoresis reveals exon 2 skipping both when an hnRNPF/H motif is introduced and when the ESE is disrupted by introduction of c.37_38GG>CC. (TIF) [file pgen.1006039.s004.tif]

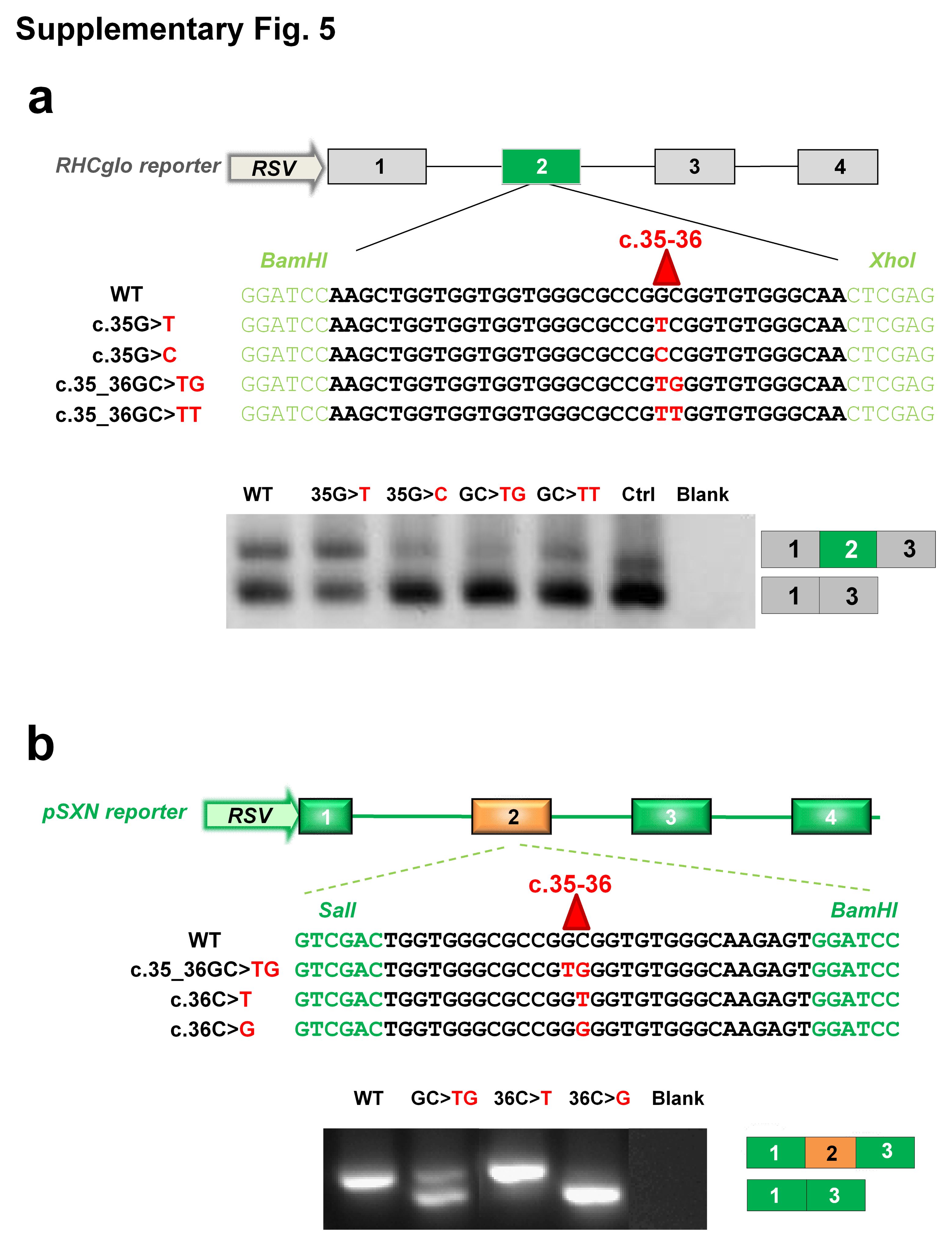

Supplement: S5 Fig — (a) Schematic overview of the RHCglo splicing reporter and construct used in this study, harboring either HRAS wild type sequence or c.35G>T, c.35G>C, c.35_36GC>TG or c.35_36GC>TT mutant sequences (top). The second exon in the RHCglo splicing reporter is immediately flanked upstream and downstream by the last and first 91 and 73 nucleotides of human β-globin intron 1, respectively. The distal upstream segment of intron 1 contains introns 1 and 3 of chicken skeletal troponin I (sTNI), and the distal downstream region of intron 2 contains the last 364 nucleotides of sTNI intron 3. Inclusion of the alternatively spliced second exon is critically dependent on the balance between ESEs and ESSs in the inserted sequence. PCR analysis of splicing of RHCglo-HRAS constructs in HepG2 cells (bottom). (b) Schematic overview of the pSXN splicing reporter and construct used in this study, holding either HRAS wild type sequence c.35_36GC>TG or c.36C>T or c.36C>G mutated sequences (top). The pSXN reporter contains an artificial small (34 bp) exon positioned between β-actin exon 1 and exon 2 as well as downstream exon 18 from cTNT. The natural sequence of β-actin intron 1 is inserted on both sides of the middle exon. The reporter contains flanking Sall and BamHI restriction sites for cloning. PCR analysis of splicing of pSXN-HRAS minigenes in HepG2 cells (bottom). (TIF) [file pgen.1006039.s005.tif]

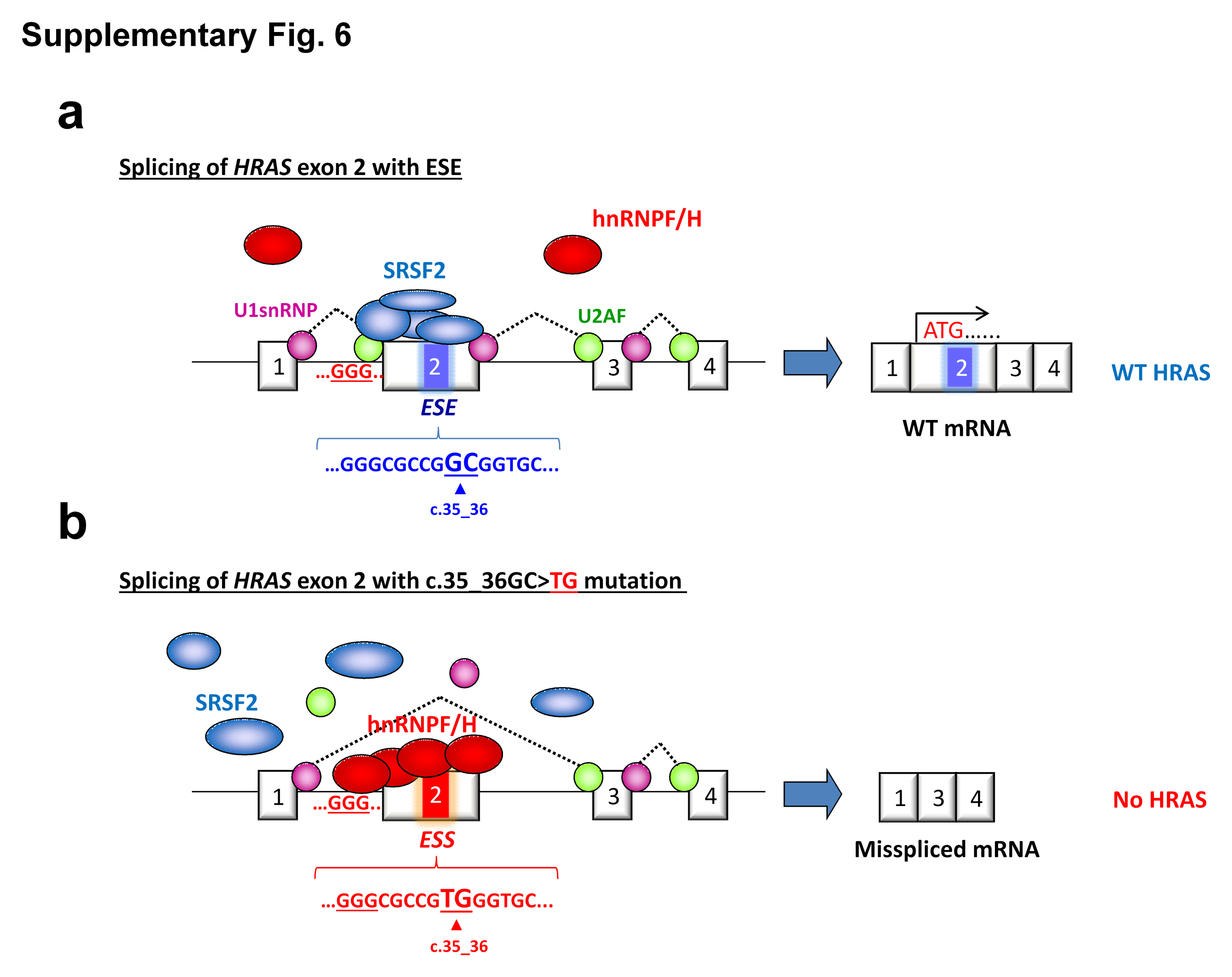

Supplement: S6 Fig — (a) Splicing of HRAS exon 2 depends on SR-proteins like SRSF2 to be recognized by the spliceosome due to a suboptimal 3’splice site. Binding of SRSF2 within exon 2 promotes its inclusion in the HRAS mRNA. (b) The c.35_36GC>TG mutation in exon 2 disrupts the ESE and creates an ESS, which binds hnRNPF/H and thereby excludes inclusion of exon 2 in the mRNA, since it cannot be recognized by the spliceosome. (TIF) [file pgen.1006039.s006.tif]

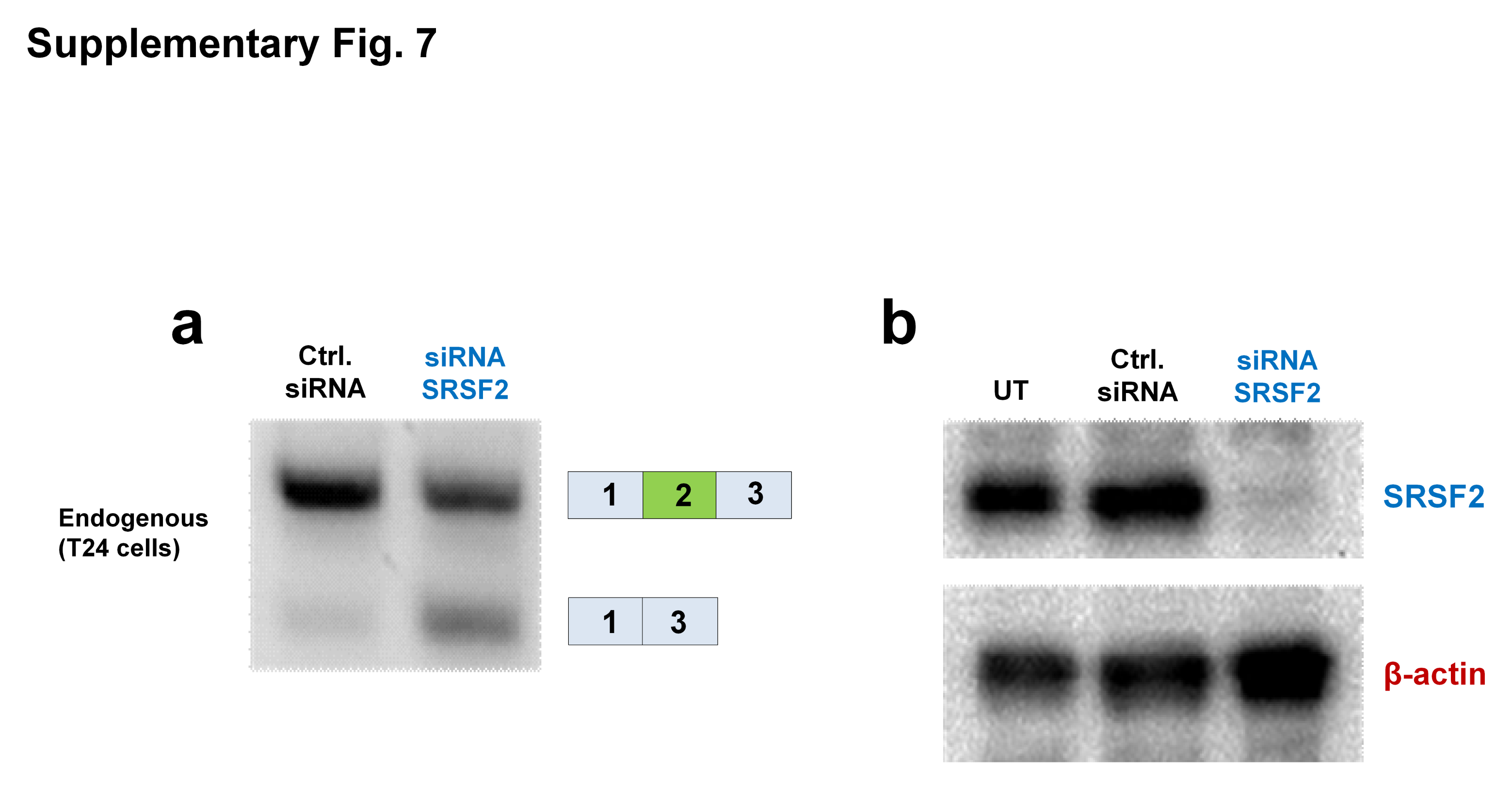

Supplement: S7 Fig — (a) PCR analysis of HRAS exon 2 skipping reveals skipping in the context of SRSF2 knock down. (b) Western blot analysis confirmed reduced levels of SRSF2 protein after knock down. β-actin was used as a control. (TIF) [file pgen.1006039.s007.tif]

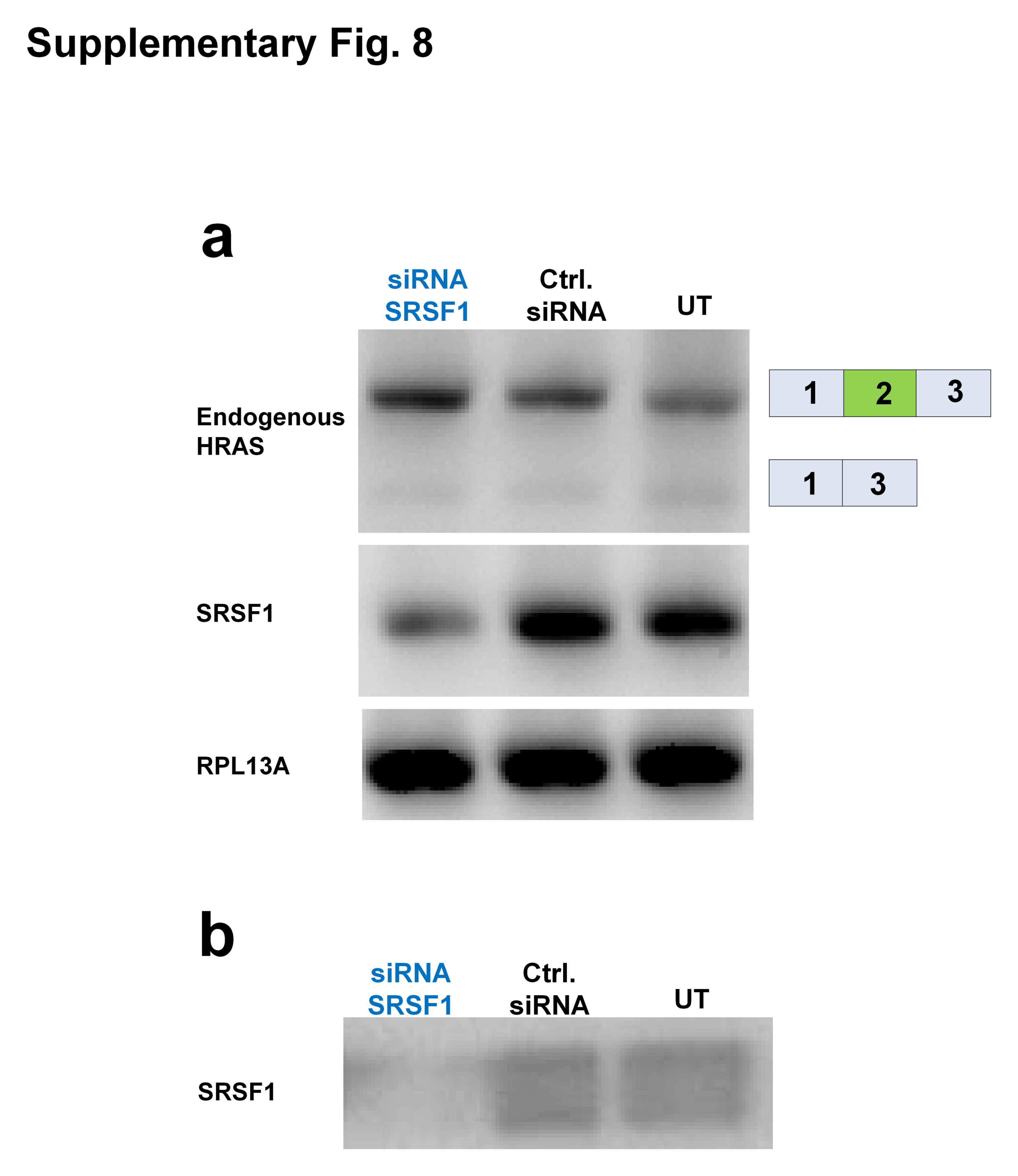

Supplement: S8 Fig — (a) PCR analysis reveals no effect on splicing of HRAS exon 2 after SRSF1 knock down. (b) Western blot analysis confirmed reduced levels of SRSF1 protein after knock down. (TIF) [file pgen.1006039.s008.tif]
